# Comparison of Statistical Algorithms for the Detection of Infectious Disease Outbreaks in Large Multiple Surveillance Systems

**DOI:** 10.1371/journal.pone.0160759

**Published:** 2016-08-11

**Authors:** Doyo G. Enki, Paul H. Garthwaite, C. Paddy Farrington, Angela Noufaily, Nick J. Andrews, Andre Charlett

**Affiliations:** 1 Medical Statistics Group, Plymouth University Peninsula Schools of Medicine and Dentistry, Plymouth, United Kingdom; 2 Department of Mathematics and Statistics, The Open University, Milton Keynes, United Kingdom; 3 Warwick Medical School, Warwick University, Coventry, United Kingdom; 4 Public Health England, London, United Kingdom; Public Health Agency of Canada, CANADA

## Abstract

A large-scale multiple surveillance system for infectious disease outbreaks has been in operation in England and Wales since the early 1990s. Changes to the statistical algorithm at the heart of the system were proposed and the purpose of this paper is to compare two new algorithms with the original algorithm. Test data to evaluate performance are created from weekly counts of the number of cases of each of more than 2000 diseases over a twenty-year period. The time series of each disease is separated into one series giving the baseline (background) disease incidence and a second series giving disease outbreaks. One series is shifted forward by twelve months and the two are then recombined, giving a realistic series in which it is known where outbreaks have been added. The metrics used to evaluate performance include a scoring rule that appropriately balances sensitivity against specificity and is sensitive to variation in probabilities near 1. In the context of disease surveillance, a scoring rule can be adapted to reflect the size of outbreaks and this was done. Results indicate that the two new algorithms are comparable to each other and better than the algorithm they were designed to replace.

## 1 Introduction

Many public health bodies tasked with surveillance of infectious diseases use statistical surveillance systems to process large quantities of data in order to detect emerging outbreaks and, if appropriate, implement control measures. For England and Wales, a laboratory-based reporting system has been the mechanism for national and regional surveillance of infectious diseases, with laboratory reports collated at the centre in London. The centre was called the Health Protection Agency (HPA) until 2013, when it became part of Public Health England (PHE). For more than twenty years the HPA/PHE used an algorithm reported in [1] to analyse these data and identify outbreaks, but work to improve the algorithm was recently undertaken and several modifications proposed [2].

The purpose of this paper is to compare the performances of two new forms of the algorithm with that of the original algorithm. Key features for an effective comparison are:

1. test data that reflects reality;
2. appropriate metrics for evaluating performance.

The primary novel features of this paper are the method of constructing test data and a scoring rule that is tailored to the task of comparing outbreak surveillance systems. The methods may be used in the evaluation of a wide variety of surveillance methods when long time series of disease counts are available.

To compare the algorithms, we are fortunate in having extensive data from PHE: weekly counts of the number of cases of each of more than 2000 infectious diseases over a twenty-year period, from 1991-2011. However, exploiting these data is not straightforward. With large outbreaks it may not be clear in which week an outbreak started or exactly when it ended. With minor abberations, it will not be known whether a slightly high value was an outbreak or whether it simply resulted from random variation. To emphasize this latter point, suppose there is a small spike in the number of cases of a disease in one particular week. One surveillance method, Method A, flags an outbreak for this week while another method, Method B, does not. Does this result suggest that Method A is better than Method B, or vice-versa? We cannot answer the question unless we know whether an outbreak really occurred in that week. Moreover, differentiating between the performances of the methods *will* involve situations where it is tricky to decide whether an outbreak occurred, as that is when methods are most likely to disagree and hence be separable. Similar problems arise when methods disagree as to when an outbreak started or ended.

Nevertheless, basing test data on real data is clearly desirable, especially as the time series for the occurrences of real diseases take extremely diverse patterns, as examples given later will illustrate. Simulated data that is generated solely from a mathematical model will not fully replicate the variety of time series that can occur, some of which have bizarre patterns or features. The approach we adopt is to take the time series of real data for each disease and separate it into two series, one giving the baseline (background) disease incidence and the other the disease outbreaks. The separation will not be perfect, but each should be a reasonable reflection of reality for the disease. We then shift one series forward by twelve months so as to destroy the close dependence between the two series at each time point, while respecting seasonal effects. The two series are then added together to form a realistic series in which we know where outbreaks have been added.

This method of constructing test time series of infectious disease data has not been proposed before. The closest related work is that of [3], [4] and [5], who each inject artificial outbreaks into time series of real surveillance data. Their aim is to evaluate surveillance methods for the fast detection of large disease outbreaks caused by bio-terrorism, which is a different context to the one we consider. In particular, outbreaks caused by bio-terrorism are rare, so past data can be equated to baseline (outbreak-free) data and, from lack of real data, the outbreaks they injected into time series had to be predominantly artificial.

A number of criteria have been proposed for evaluating the performance of outbreak detection systems; reviews may be found in [6] and [7]. It is commonly recommended that a variety of evaluation criteria should be examined to see if a surveillance method is fit for purpose [8]. Here the criteria we shall examine reflect specificity, sensitivity, time before an outbreak is detected, and the number of cases before detection. As specificity can easily be improved at the cost of poorer sensitivity, or vice-versa, combined measures that balance specificity against sensitivity are needed. ROC curves and related curves have been used for this purpose, but a separate curve would be needed for each infectious disease being monitored, and here there are a large number of diseases.

Instead, we propose the use of a scoring rule that is based on the probabilities that a surveillance method gives for ‘outbreak’ and ‘no outbreak’ each week. A novel feature of the scoring rule is that it reflects the number of cases that have been observed, giving greater weight to higher counts, regardless of whether or not there was an outbreak that week. In practice, this means that missing a large outbreak is generally penalised more than missing a small outbreak. This seems appropriate, as missing a large outbreak tends to have more adverse consequences. Also, a surveillance algorithm must sometimes miss very small outbreaks if it is to have reasonable specificity.

An important question is how to strike a balance between specificity and sensitivity. One way of partially answering this question is to consider the situation where the time series of disease occurrences is generated from a mathematical model. Then, given the data for the current week and preceding weeks, we could know, in principle, the true probability each week of ‘outbreak’ and ‘no outbreak’. A surveillance system that consistently matches these probabilities should expect to get a better score than a system that does not match these probabilities. By definition this will be the case if, and only if, the scoring rule is *strictly proper*. The scoring rule we use is strictly proper, so the expected score is maximised by giving probabilities that mirror reality. The purpose of the surveillance algorithms is to monitor a large number of diseases to detect which of them, if any, display signs of an outbreak. From this perspective, the only results of interest are those that suggest an outbreak is likely or highly likely to have arisen. Our chosen scoring rule reflects this purpose; it is asymmetric and discriminates far more between probabilities in the range 0.95–1 than in the range 0–0.9. To our knowledge, this is the first use of a proper scoring rule that is targeted to a small section of the probability range.

In Section 2 we briefly describe the outlier detection algorithms examined in this paper. In Section 3 we describe the test data used for evaluation and the procedure for deriving it from the historical time series. The motivation for the procedure and its benefits are discussed. The evaluation criteria used to compare the performance of algorithms are given in Section 4. These include the novel scoring rule. Results from the comparison of algorithms are presented in Section 5 and some concluding comments are made in Section 6.

## 2 The outbreak detection algorithms

We shall refer to the original algorithm proposed by [1] as the *HPA algorithm* because of its long-term use by the HPA. [2] examined the effects of their proposed modifications using simulated data generated from a mathematical model. Based on the results, two modified algorithms were proposed and applied to real data. These are the two other algorithms that we examine here. Following Noufaily et al., we refer to these as the *quasi-Poisson algorithm* and the *negative binomial algorithm*.

The purpose of each algorithm is to provide an exceedance score (*X*) for each disease, where this is defined as

$$\begin{array}{c} X=\frac{y_{0}-{\hat{\mu }}_{0}}{U-{\hat{\mu }}_{0}}. \end{array}$$ (1)

In this equation, *y*_0_ is the number of cases of the disease in the current week, ${\hat{\mu }}_{0}$ is its expected value, and *U* is a threshold value such that Pr(*y*_0_ > *U*) approximately equals some small value, *α* say, if there is no outbreak of the disease in the current week. (Typically *α* is set equal to 0.005 or, less commonly, 0.025.) Diseases with *X* ≥ 1 are flagged for more detailed investigation and a list is produced that ranks the flagged diseases in the order given by their exceedance scores. For each disease, the algorithms also produce a *p*-value from a hypothesis test that there is no outbreak of the disease in the current week.

The algorithms all fit a weighted quasi-Poisson model to a time series of counts of disease cases over the preceding five years, allow the expected count to follow a linear trend over time with seasonal variation, and down-weight counts that are unusually high as the aim is to model the underlying baseline (outbreak-free) time series. Some details are given in Appendix A and further detail may be found in [1] and [2].

The only difference between the negative-binomial algorithm and the quasi-Poisson algorithm is that the former uses a negative binomial distribution to calculate the threshold *U* while the latter (and also the HPA algorithm) use a normal approximation. The differences between the HPA algorithm and these other two algorithms are greater. The HPA algorithm only includes a linear time trend if there is evidence that there is a trend and uses different criteria for down-weighting past values that are high. Also, to handle seasonality it restricts the data it uses from any year to a seven-week window centred on the current week, while the other algorithms use the full data and model seasonality using a 10-level factor. From its original design, the HPA algorithm does not flag a disease as a potential outbreak unless the total count in the past four weeks exceeds 4. Noufaily et al. considered forms of negative-binomial and the quasi-Poisson algorithms that adopted this policy and those are the forms used here, as small isolated counts of a very rare disease should not, in general, be flagged as aberrant. (Although any case of certain dangerous diseases always requires investigation.)

## 3 The test data

From the simulation study that they conducted, Noufaily et al. concluded that the false-positive rate given by the HPA algorithm is too high, primarily due to excessive down-weighting of high baseline values and reliance of too few baseline weeks. They applied the algorithms of interest here to real data from PHE for the year 2011 and found that the two modified algorithms flagged high values at a much lower rate than the HPA algorithm. However, the specificity and sensitivity of algorithms could not be determined because of the limitations of using real data: the precise occurrences and sizes of outbreaks are not known. Here we start with historical time series of real disease counts and from them construct realistic time series in which the details of outbreaks are known.

The data are from PHE for the years 1991-2011 and relate to 3,303 distinct types of infectious organism whose occurrence frequencies range across six orders of magnitude. These data have been analysed by [9], who examine its characteristics and note a diversity of seasonal patterns, trends, artifacts and extra-Poisson variation. Following [9], data from the last 26 weeks of 2011 will not be used so as to mitigate the effects of delays in data processing at the end of the series. (Reporting delays are an issue with laboratory data of the form available to us. Methods to incorporate the effects of reporting delays in statistical surveillance systems have been the subject of separate research [10]). Some organisms that were identified towards the end of the study period would not have been identified by the tests that were performed a decade or so earlier. In line with Enki et al., all leading sequences of zeros are recoded as missing values and, to reduce the selection bias this introduces, the first non-zero count is reduced by one. There are 999 diseases for which there are less than eight years data and these are omitted from our analysis, reducing the number we examine to 2304.

**Fig 1:**
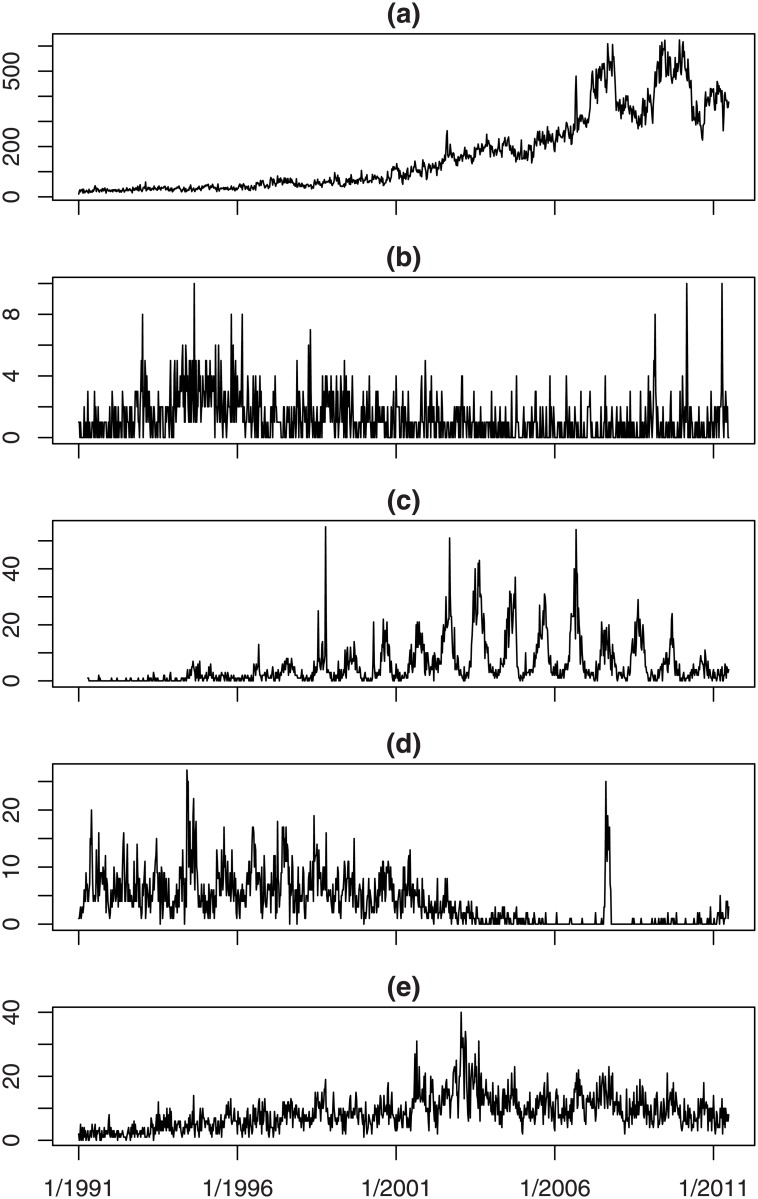
Time series for selected organisms.

The diversity in the time series of different diseases is illustrated in Fig 1, where plots of the weekly counts for five selected organisms are given. The organisms vary considerably in their weekly counts, with plot (a) (*Streptococcus coagulase negative*) showing counts that sometimes exceed 600, while the counts in plot (b) (*Schistosoma haematobium*) never exceed 10. Shapes also vary. Plot (a) shows a clear trend, plot (b) is flat with a number of one-week spikes, plot (c) (*Salmonella enteritidis PT21*) shows marked seasonality and plot (d) (*Campylobacter coli*) and plot (e) (*Acinetobacter SP*) show both seasonality and trend.

The following steps were followed for each organism to separate its time series of weekly counts into one time series of baseline data (series *A*) and another of outbreaks (series *B*). The procedure is iterative and starts with *A* equated to the observed time series, {*y_i_*, *i* = 1, 2, …}, where *y*_*i*_ is the count in week *t*_*i*_ for the organism of current interest.

*Step 1*. As noted in Section 2, none of the three algorithms will flag a disease as a potential outbreak unless at least five cases of the disease have been reported in the past four weeks. In line with this, the time series is examined to see whether it includes at least one sequence of four weeks in which there were five or more cases of the disease. If not, then the series will not help differentiate between the algorithms, so it is omitted from all analyses and no further steps are performed for it.

*Step 2*. A generalised linear model (GLM) or generalised additive model (GAM) is fitted to *A* using the mgcv package in *R*. In either case, a quasi-Poisson model with a log link function is used and a seasonal covariate factor is included if there is evidence (at the 5% significance level) that its inclusion improves model-fit. The seasonal factor has 12 levels, one for each month. A GLM is fitted if there is no evidence (at the 5% significance level) of a linear trend over time; otherwise a GAM with a smoothed time trend is fitted. Output from the GLM or GAM includes fitted values giving the weekly expected disease counts and an estimate of the dispersion parameter, *ϕ*, that is constrained to $\hat{\phi }\geq 1$. The time series in *A* is long (weekly counts over at least an 8-year period) and the models are not complex, so the risk of overfitting is small and any overfitting would have little effect.

*Step 3*. We next determine whether any weekly counts in *A* should be classified as extreme. Let $y_{i}^{*}$ denote the value in *A* for week *t*_*i*_. [Unless the original value in *A* for week *t*_*i*_ has been replaced (c.f. step 4), $y_{i}^{*}=y_{i}$.] The underlying base rate for that week is taken as the expected count, ${\hat{\mu }}_{i}$, and if there is no outbreak, an estimate of the variance of the count is $\hat{\phi }{\hat{\mu }}_{i}$.

Any weekly count of two or less is classified as ‘not extreme’. For other weeks:

1. We first classify $y_{i}^{*}$ as extreme if it exceeds ${\hat{\mu }}_{i}+2{\left(\hat{\phi }{\hat{\mu }}_{i}\right)}^{1/2}$.
2. For each of the weeks whose counts are ‘extreme’, we check if the counts of the adjacent weeks are also extreme, but use less stringent criteria because outbreaks commonly last more than a week. We classify $y_{i}^{*}$ as extreme if its value exceeds ${\hat{\mu }}_{i}+1.5{\left(\hat{\phi }{\hat{\mu }}_{i}\right)}^{1/2}$ and it is next to a value that is extreme.

Although extreme values may arise from a number of reasons, we refer to them collectively as ‘outbreaks’ because they should all be flagged by outbreak detection algorithms, whose aim is to detect cases of a disease that are not part of the baseline series.

*Step 4*. We note the weeks in which values have been classified as extreme. They are then dropped from *A* and replaced. If $y_{i}^{*}$ is dropped, it is replaced with a random value from a negative binomial distribution with mean ${\hat{\mu }}_{i}$ and variance $\hat{\phi }{\hat{\mu }}_{i}$, under the constraint that the new value must be less than ${\hat{\mu }}_{i}+1.5{\left(\hat{\phi }{\hat{\mu }}_{i}\right)}^{1/2}$.

*Step 5*. We then return to step 2 and repeatedly cycle through steps 2–4 until step 3 identifies none of the values in *A* as extreme.

*Step 6*. When this step is reached, series *A* contains the final estimate of the baseline data. The difference in weekly values between the real data and *A* gives *B*, our weekly time series of outbreaks.

An R program used in the implementation of the above steps is provided as supporting information [S1 R.Codes].

**Fig 2:**
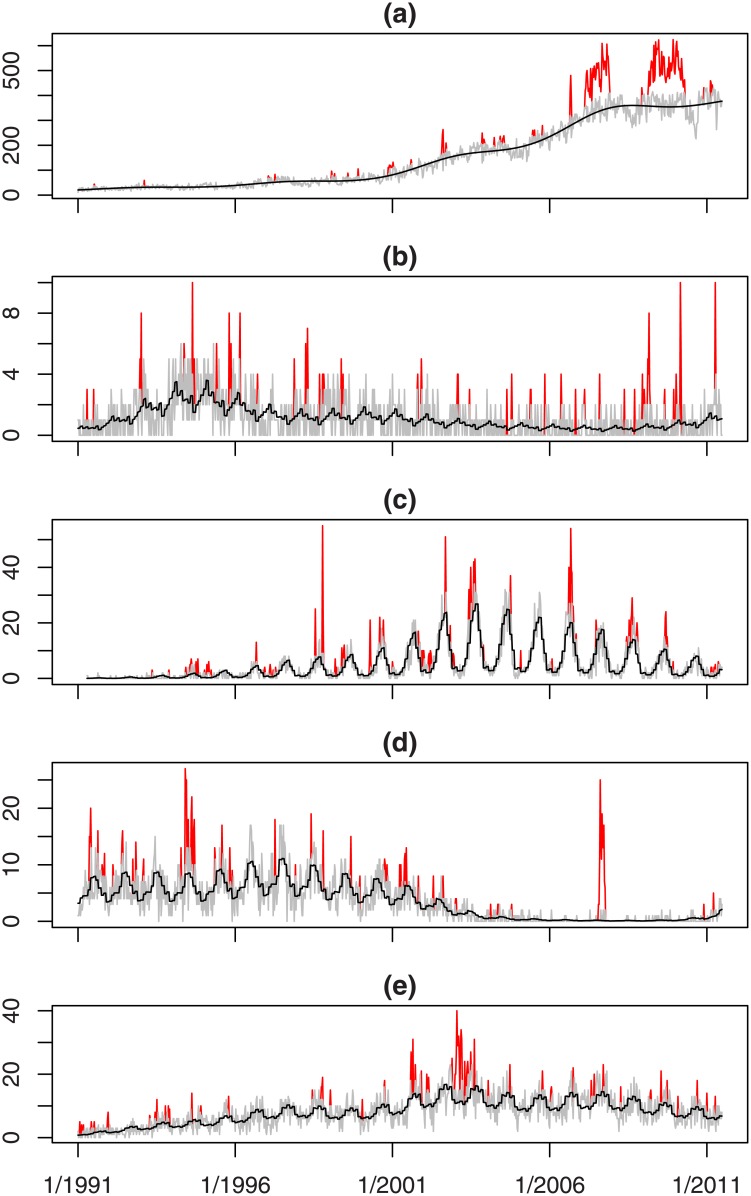
Time-series separated into baseline (grey line) and outbreaks (darker/red lines) for selected organisms; continuous black line shows the baseline expected counts when there is no outbreak.

Fig 2 takes the time series shown in Fig 1 and separates each of them into series *A* and series *B*. The lighter (grey) line in each plot is *A* and the non-zero values in *B* are shown by the darker (red) lines. The black line shows the baseline expected counts when there is no outbreak. The baseline is meant to follow any trend or seasonality in series *A* and in the plots it appears to do this well.

We want time series in which we know where outbreaks have occurred. One approach is to simulate outbreaks from a mathematical model and add them to series *A*, so that the baseline incidence mirrors reality. Another approach is to add series *B* to baseline data simulated from a mathematical model, so that the outbreak data mirrors reality. With both these approaches, the outbreaks and baseline data are independent so it is clear that outbreaks have been injected into a baseline. Also, we know when outbreaks have been injected and their sizes.

In contrast, the following criticisms are valid if we simply add *A* to *B* to form the test time series.

1. Independent outbreak data have not been injected into baseline data. Rather, the original data series has simply been reconstructed.
2. The method of identifying extreme counts (so as to form *B*) uses only slightly more information than the outbreak detection algorithms being tested. (They only differ as to whether future data are used to decide if the count in the week of current interest is extreme.) Consequently, when this method and, say, the HPA algorithm disagree as to whether a count in a particular week is extreme, it is not clear which should be taken as correct. That is, we do not know when outbreaks have occurred with sufficient certainty.

Nevertheless, using *A* to model baseline incidence and *B* to model outbreaks is attractive, as this makes full use of the data. The solution that we adopt is to shift *B* forward by one year, giving a new series (series *C* say) that has the same annual timing of any seasonal pattern that was present in *B*. We then create the series of test data by adding *A* to *C* for the 19.5 years where these two time series overlap. The relationship between contemporaneous elements of *C* and *A* is weak (the element in *C* is from the previous year), so it is reasonable to treat the addition of *C* to *A* as the injection of outbreaks into unrelated baseline data. Weeks in which *C* is non-zero are classified as the weeks in which outbreaks occur. As long as *A* is a realistic representation of a baseline weekly disease count and *B* is a realistic sequence of outbreaks of the disease, then *A* + *C* should be a realistic time series on which to test outbreak detection algorithms. Moreover, we have more information about the occurrence of outbreaks than is available to the algorithms under test. In particular, we can be certain that outbreaks have been injected in those weeks where the count in the test data is higher than in the real data. This covers the great majority of classified outbreaks: the count is higher in the test data in 94% of the weeks for which series *C* is non-zero.

**Fig 3:**
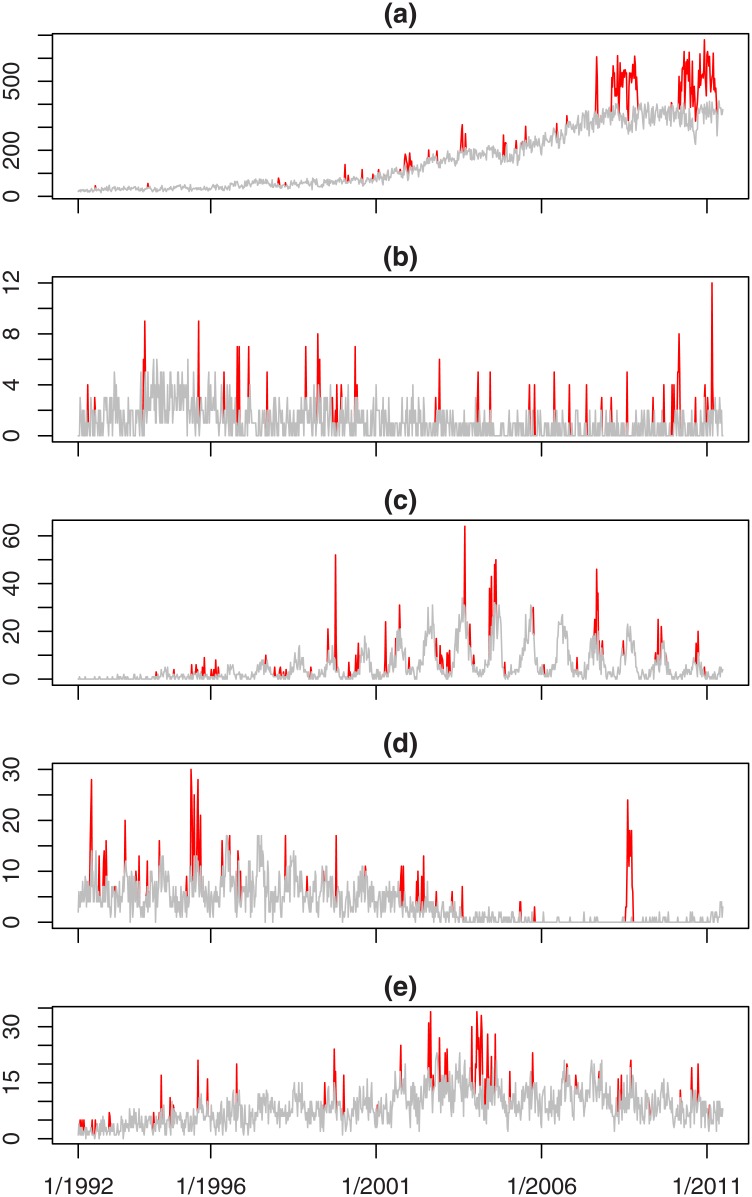
The test series for selected organisms showing the baseline (grey line) and injected outbreaks (darker/red lines).

Fig 3 shows the test series given by *A* + *C* for each of the diseases considered in Figs 1 and 2. The lighter (grey) line shows the baseline counts given by series *A* and the darker (red) lines show the injected outbreaks given by series *C*. In each plot their combination (*A* + *C*) looks as if the corresponding time series in Fig 1 has simply been moved along by 12 months. Thus *A* + *C* does give realistic time series of disease counts. The darker (red) lines are fairly sparse so *C* has non-zero values for comparatively few weeks. Thus most of the counts in Fig 1 have not been moved up 12 months to form Fig 3—this only appears to be the case because the eye-catching features (the outbreaks) have been identified and translated by 12 months. Thus the time series in Fig 3 meet the requirements of being realistic with outbreaks that have been injected.

## 4 Evaluation measures

### 4.1 *Standard Metrics*

Algorithms were applied to the test data of each organism and they classified the count in each week as extreme/not extreme, which we equate to outbreak/no outbreak. The following metrics will be used to evaluate an algorithm’s performance. They relate to specificity, sensitivity and timeliness.

*False positive rate* (FPR). This is the proportion of weeks in which a count was flagged as extreme but, in fact, there was no outbreak. A low FPR corresponds to good specificity.

*Probability of detection* (POD). Each sequence of weeks in which series *C* has non-zero values defines the timing of one outbreak. An outbreak is deemed to be detected if the algorithm flags a count as extreme at least once during the outbreak’s duration. POD is the proportion of outbreaks that are detected; a high POD corresponds to good sensitivity.

*Scaled probability of detection* (ScPOD). From a practical viewpoint, missing large outbreaks matters more than missing small outbreaks. This metric reflects the proportion of outbreaks detected and their sizes. Let *n*_*j*_ denote the number of cases in the *j*th outbreak (obtained from *C*). Suppose there are *N* outbreaks and that the algorithm detected the *j*th outbreak if *j* ∈ *J*, where *J* is a set of positive integers. We define the scaled probability of detection as

$$\begin{array}{c} \text{ScPOD}\;=\;\sum_{j\in J}n_{j}/\sum_{j=1}^{N}n_{j}. \end{array}$$ (2)

*Average time before detection* (ATBD). For a specified set of N* outbreaks, this is the average number of weeks between an outbreak starting and its detection (excluding the week of detection). The set of outbreaks will consist of outbreaks of specified sizes but will exclude outbreaks that were not detected. Let $m_{j}^{*}$ denote the number of weeks before an outbreak’s detection, where *j* ∈ J* if $m_{j}^{*}$ relates to an outbreak in the set. Then

$$\begin{array}{c} \text{ATBD}\;=\;\sum_{j\in J^{*}}m_{j}^{*}/N^{*}. \end{array}$$ (3)

The overall ATBD is the ATBD for the set of all outbreaks that were detected. A good algorithm will not only detect a large proportion of outbreaks (which POD and ScPOD measure), but will also detect them quickly, which this metric measures.

*Relative size before detection* (RSBD). The timeliness of a surveillance system also depends on the number of outbreak cases that occur before an outbreak is detected. To obtain a measure that is not dominated by common diseases, we relate this number to baseline values. Let *v*_*j*_ denote the number of cases in the *j*th outbreak during the weeks before its detection (given by series *C*) and let *u*_*j*_ denote the number of baseline cases during those weeks (given by series *A*). If *u*_*j*_ equates to an average of less than two cases per week, we increase it so that it equals two cases per week, so that uncommon diseases do not have a disproportionate affect. We define the relative size before detection as

$$\begin{array}{c} \text{RSBD}\;=\;\sum_{j\in J^{*}}\left(v_{j}/u_{j}\right)/N^{*}. \end{array}$$ (4)

### 4.2 *A Tailored Scoring Rule*

Scoring rules gained prominence in the context of weather forecasting. For some daily forecasts of whether it will rain, meteorologists gave a “probability of precipitation” and scoring rules were used to evaluate the accuracy of these probabilities. One purpose of a scoring rule is to encourage honesty. A scoring rule is said to be *proper* if a person can maximize his (or her) expectation of his score by reporting his true beliefs and it is said to be *strictly* proper if a person can *only* maximize his expectation of his score by reporting his true beliefs.

A scoring rule should be proper if it is to play a role in evaluating surveillance methods for disease outbreaks. To illustrate this, we consider a scoring rule that is not proper, though at first sight it seems a sensible rule. Specifically, suppose that, when a surveillance method estimates *p*_*i*_ as the probability of an outbreak in week *t*_*i*_, then its score is given by:

$$\begin{array}{c} \text{score}=\left\{\begin{array}{cc} p_{i} & \text{if}\;\text{an}\;\text{outbreak}\;\text{has}\;\text{occurred}\\ 1-p_{i} & \text{if}\;\text{no}\;\text{outbreak}\;\text{has}\;\text{occurred.} \end{array}\right. \end{array}$$ (5)

This is called the linear scoring rule. It seems a reasonable rule as it assigns a score equal to the estimated probability of the event that occurred.

Now suppose we are comparing two surveillance methods that calculate each week the probability that an outbreak has occurred. Suppose $p_{i}^{*}$ is a realistic estimate of this probability in week *t*_*i*_. (In a simulation study we could calculate $p_{i}^{*}$, as the mechanism for generating the number of cases in a week is known.) Suppose further that one surveillance method always estimates $p_{i}^{*}$ accurately while the second method is biased and gives probabilities that are too extreme: when $p_{i}^{*}>0.5$ the probability it gives is greater than $p_{i}^{*}$ while, when $p_{i}^{*}<0.5$, the probability it gives is less than $p_{i}^{*}$. Clearly a decent scoring rule should give a better long-term average score to the accurate surveillance method, rather than the biased method. However, if a surveillance method gives *p*_*i*_ as the probability of an outbreak in week *t*_*i*_, then the expected score that it receives from the linear scoring rule, E(*S*_*i*_) say, is

$$\begin{array}{c} \text{E}\left(S_{i}\right)=p_{i}^{*}p_{i}+\left(1-p_{i}^{*}\right)\left(1-p_{i}\right). \end{array}$$ (6)

As $d\text{E}\left(S_{i}\right)/dp_{i}=2p_{i}^{*}-1$, it follows that the biased surveillance method has a higher expected score than the accurate method, both when $p_{i}^{*}>0.5$ and when $p_{i}^{*}<0.5$. This is clearly inappropriate and is the type of error that cannot arise with a proper scoring rule: with a proper scoring rule, by definition, the expected score is maximized by setting *p*_*i*_ equal to $p_{i}^{*}$, as that is the realistic (honest) estimate of the probability of an outbreak.

Strictly proper scoring rules provide a natural balance between sensitivity and specificity. When there is an outbreak in week *t*_*i*_, a large value of *p*_*i*_ corresponds to good sensitivity and yields a good score. When no outbreak has occurred, a low value of *p*_*i*_ corresponds to good specificity and this also yields a good score. If it is strictly proper, the scoring rule ensures that the expected score is maximised by giving a realistic value to *p*_*i*_ at each time point, thus balancing the contrasting needs for good sensitivity (set *p*_*i*_ high) and good specificity (set *p*_*i*_ low).

The most common scoring rules are the logarithmic, quadratic and spherical rules. These are not well-suited to the present context because we require a scoring rule that is insensitive to values of *p*_*i*_ in the range 0 to 0.9, and very sensitive to variation in *p*_*i*_ in the range 0.95 to 1—when a disease has a value of *p*_*i*_ in the former range (below 0.9), no action will be taken to investigate whether there is an outbreak of the disease (unless the seriousness of a disease means that all occurrences are investigated), while the impetus to investigate increases substantially as *p*_*i*_ increases from 0.95 to 1. A flexible choice of scoring rules is based on the family of beta distributions [11]:

$$\begin{array}{c} \text{score}=\left\{\begin{array}{cc} g_{i}F_{1}\left(p_{i}\right) & \text{if}\;\text{an}\;\text{outbreak}\;\text{occurred}\;\text{in}\;\text{week}\;t_{i}\\ g_{i}\left\{1-F_{2}\left(p_{i}\right)\right\} & \text{if}\;\text{no}\;\text{outbreak}\;\text{occurred}\;\text{in}\;\text{week}\;t_{i}\text{,} \end{array}\right. \end{array}$$ (7)

where *F*_1_ and *F*_2_ are the cumulative distribution functions of beta(*a*, *b* + 1) and beta(*a* + 1, *b*) probability distributions, respectively, and *g*_*i*_ does not influence *p*_*i*_. The scoring rule is asymmetric if *a* ≠ *b* and strictly proper for any *a*, *b* > −1 [12]. Here we set *a* = 40 and *b* = 0.5. Table 1 lists the scores that this rule gives for a variety of values of *p*_*i*_ when *g*_*i*_ = 1. It can be seen that all values of *p*_*i*_ ∈ (0, 0.9) give similar scores, while scores change substantially as *p*_*i*_ increases from 0.95 to 1.0. The occurrence of an outbreak (rather than no occurrence) only gives the higher score when *p* exceeds 0.985. If an alarm should be sounded when an outbreak has likely occurred, then sounding it for values of *p* above 0.985 might make a suitable threshold, as the alarm should only be raised when there is clear evidence that an outbreak has occurred.

**Table 1:** Scores given to the probability of outbreak (*p*) using beta(40, 1.5) and beta(41, 0.5) distributions for *F*_1_ and *F*_2_.

	Outbreak	No outbreak		Outbreak	No outbreak
*p*	*F*_1_(*p*)	1 − *F*_2_(*p*)	*p*	*F*_1_(*p*)	1 − *F*_2_(*p*)
0.0	0.0	1.0	0.99	0.847	0.635
0.9	0.037	0.997	0.995	0.940	0.477
0.95	0.248	0.959	0.999	0.994	0.225
0.97	0.484	0.885	0.9995	0.998	0.160
0.98	0.653	0.801	1.0	1.0	0.0

In judging the performance of algorithms, the most important weeks are those when the count of an organism is noticeably higher than usual for the time of year. Those are the weeks in which an algorithm might incorrectly flag an outbreak when one has not occurred, or when one algorithm might correctly flag an outbreak while another algorithm does not. Also, if a large outbreak has occurred, we would like it to be flagged very clearly. In contrast, missing very small outbreaks (perhaps of just two or three cases) seems inevitable if the false discovery rate is to be kept low, so missing large outbreaks should be penalised more. Thus, in Eq (7) we want *g*_*i*_ to reflect the size of an outbreak while

1. *g*_*i*_ should not equal 0 when there is no outbreak—weeks with no outbreak are less important but not unimportant.
2. The value of *g*_*i*_ should give no information about outbreaks that is not readily available to the outbreak detection algorithm. This is necessary for the validity of the scoring rule—otherwise it might be possible to improve an algorithm (when judged by expected score) by exploiting the value of *g*_*i*_.

To illustrate the relevance of (b), note that the scoring rule would be proper if *g*_*i*_ took a randomly generated value, even if that value varied from week to week. The scoring rule would still be proper if *g*_*i*_ were set equal to *y*_*i*_, the number of cases in the current week, as that information is available to the outbreak detection algorithms (and used by them). However, some choices of *g*_*i*_ could change an algorithm’s optimal approach to estimating *p*_*i*_. As an extreme example, if *g*_*i*_ were set equal to the size of the outbreak in the current week, equalling 0 when there is no outbreak, then setting *p*_*i*_ equal to 1 for all *i* maximises the expected score.

The approach we advocate is to set *g*_*i*_ equal to max[0, (*y*_*i*_ − *η*_*i*_)^1/2^], where *η*_*i*_ is a low estimate of the baseline count for week *i*. In implementing the scoring rule, we take the corresponding week in each of the previous five years and set *η*_*i*_ equal to the smallest number of cases in any one of those weeks. (For example, if week *t*_*i*_ is the first week of August 2011, then *η*_*i*_ is taken as the smallest number of cases in the first week of August in the years 2006 to 2010). Thus *g*_*i*_ is proportional to the square-root of a high estimate of the outbreak size. This gives greater importance to large outbreaks, while *g*_*i*_ will usually also be non-zero in weeks without outbreak. We use max[0, (*y*_*i*_ − *η*_*i*_)^1/2^], rather than max[0, *y*_*i*_ − *η*_*i*_], so that a few common diseases do not dominate the overall scores of algorithms.

While the above form of scoring rule is useful for evaluating surveillance methods, it cannot be applied to weather forecasting because of intrinsic differences between the two contexts. With surveillance methods a quantity is observed that relates to the magnitude of an outbreak if an outbreak has occurred, but the question of whether an outbreak has occurred is still of interest. In weather forecasting this does not happen when, say, the forecast is giving the probability of rain: if the quantity of rain that has fallen in a day is known, then we know whether it has rained. Consequently, in weather forecasting other approaches have been proposed to take the quantity of rainfall into account in evaluating rainfall forecasts [13, 14]. However, the methods do not give proper scoring rules.

To apply the scoring rule to an algorithm, the algorithm’s value for *p*_*i*_ must be calculated for each week. If there were four or fewer cases of disease in the last four weeks then, by assumption, there is no outbreak in the current week and *p*_*i*_ is set equal to 0. Similarly, we set *p*_*i*_ equal to 0 in weeks where the observed count is less than the number the algorithm expects when there is no outbreak. For other weeks, let *B*_*i*_ denote the event that there is an ongoing outbreak of the disease under consideration at time *t*_*i*_ and let $B_{i}^{c}$ denote the (complementary) event that there is no outbreak at time *t*_*i*_. To obtain *p*_*i*_, we calculate (i) $P(y_{i}|B_{i}^{c},H_{i})$, (ii) *P*(*B*_*i*_|*H*_*i*_), and (iii) *P*(*y*_*i*_|*B*_*i*_, *H*_*i*_), where *H*_*i*_ denotes the historical data available at time *t*_*i*_. Then *p*_*i*_ is given by Bayes theorem,

$$\begin{array}{c} p_{i}=P\left(B_{i}|y_{i},H_{i}\right)=\frac{P\left(y_{i}|B_{i},H_{i}\right)\times P\left(B_{i}|H_{i}\right)}{P\left(y_{i}|B_{i},H_{i}\right)\times P\left(B_{i}|H_{i}\right)+P\left(y_{i}|B_{i}^{c},H_{i}\right)\times P\left(B_{t}^{c}|H_{i}\right)}. \end{array}$$ (8)

The outbreak detection algorithms were designed to evaluate the probability that the count *Y*_*i*_ is greater than a threshold value, *U*, when there is no outbreak. Hence we can obtain $P(Y\geq y_{i}|B_{i}^{c},H_{i})$ and $P(Y\geq y_{i}+1|B_{i}^{c},H_{i})$ from the algorithm under consideration and, for (i), equate $P(y_{i}|B_{i}^{c},H_{i})$ to their difference. For (ii), we equate *P*(*B*_*i*_|*H*_*i*_) to the proportion of weeks in the series that are classified as ‘extreme’ by the algorithm. It seems prudent to anticipate an outbreak in at least one week in twenty years, so we set *P*(*B*_*i*_|*H*_*i*_) equal to 0.001 if it is less than that value.

Quantity (iii), *P*(*y*_*i*_|*B*_*i*_, *H*_*i*_), is trickier to determine. For each organism, we first identify the weeks for which the algorithm flags an abberation or outbreak (hence called outbreak-weeks). If there were more than five outbreak weeks then:

1 For both outbreak and non-outbreak weeks we calculate the standardised count,
  $$\begin{array}{c} SC\left(y_{i}|H_{i}\right)=\frac{y_{i}^{2/3}-{\hat{\mu }}_{i}^{2/3}}{{\left\{\text{var}\left(y_{i}^{2/3}-{\hat{\mu }}_{i}^{2/3}\right)\right\}}^{1/2}}, \end{array}$$ (9)
  where *y*_*i*_ is the observed count that week and ${\hat{\mu }}_{i}$ is the (baseline) count predicted by the algorithm. Both ${\hat{\mu }}_{i}$ and $\text{var}(y_{i}^{2/3}-{\hat{\mu }}_{i}^{2/3})$ are used in calculating exceedance scores, so they are readily available from each algorithm.
2 We select the outbreak weeks and fit a log-normal distribution to the standardised counts of those weeks.
3 For all weeks (except when *p*_*i*_ has not been set to 0), we equate *P*(*y*_*i*_|*B*_*i*_, *H*_*i*_) to the difference between *P*{*ξ* ≥ *SC*_*i*_(*y*_*i*_)} and *P*{*ξ* ≥ *SC*_*i*_(*y*_*i*_ + 1)}, where *ξ* is a variable that has the log-normal distribution fitted in (ii).

If there were five or fewer outbreak weeks then:

4 Taking just the outbreak weeks, a Poisson distribution is fitted to the counts above baseline, $y_{i}-{\hat{\mu }}_{i}$, with the Poisson parameter equated to a constant (*λ*) multiplied by the standard deviation of the baseline count at *t*_*i*_, (*ϕμ*_*i*_)^1/2^. The maximum likelihood estimate of *λ* is $\hat{\lambda }=\sum_{i}\left(y_{i}-{\hat{\mu }}_{i}\right)/\sum_{i}{\left(\hat{\phi }{\hat{\mu }}_{i}\right)}^{1/2}$, where the summations cover the outbreak weeks.
5 For all weeks (except when *p*_*i*_ = 0), *P*(*y*_*i*_|*B*_*i*_, *H*_*i*_) is equated to the probability of $y_{i}-{\hat{\mu }}_{i}$ under a Poisson distribution with mean $\hat{\lambda }{\left(\hat{\phi }{\hat{\mu }}_{i}\right)}^{1/2}$.

## 5 Results

There are 2304 diseases for which we have eight years or more of continuous data. Of these diseases, 1345 showed no sequence of four weeks in which there were five or more cases of the disease and so have no count that is classified as an outbreak by any of the algorithms. Those diseases are not considered further in our analyses.

**Fig 4:**
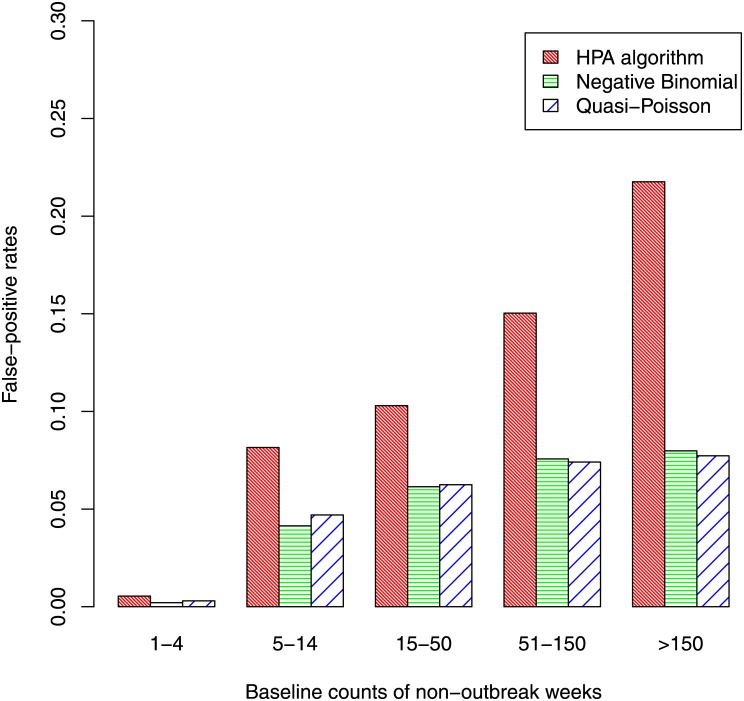
False-positive rates.

**Fig 5:**
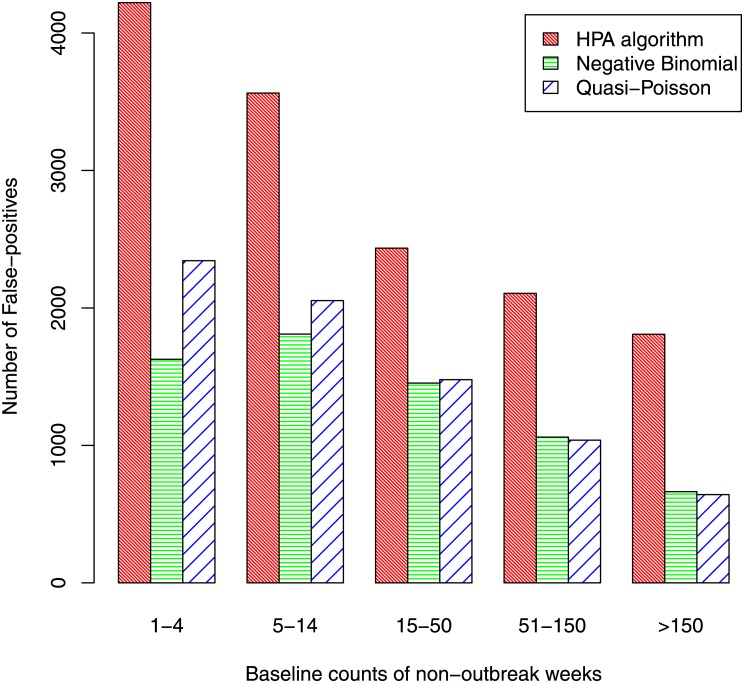
Number of false-positives by baseline count.

The false positive rates for the other 959 diseases are shown in Fig 4. The rates vary greatly according to the baseline disease count (the values in series *A*) and were calculated for five groups: counts of 1–4, 5–14, 15–50, 51–150, and over 150. Fig 4 shows that the false-positive rate increases substantially as the baseline count increases—clearly large baseline counts make the identification of outbreaks more problematic, as might be expected. Although the first group has very low false-positive rates, the rates in this group are important because it is much the largest group and, in terms of actual numbers rather than rates, there are more false positives in this group than any other. The number of false-positives in each group are shown in Fig 5. Comparing algorithms, the false-positive rates (and the number of false-positives) are typically about twice as high for the HPA algorithm as for the negative binomial and quasi-Poisson algorithms. The false positive rates for these latter two algorithms are fairly similar, although the negative-binomial performed a little better than the quasi-Poisson when the baseline count was small. When groups are combined, the overall false-positive rate was 1.63% for the HPA algorithm, 0.76% for the negative binomial algorithm and 0.87% for the quasi-Poisson.

**Fig 6:**
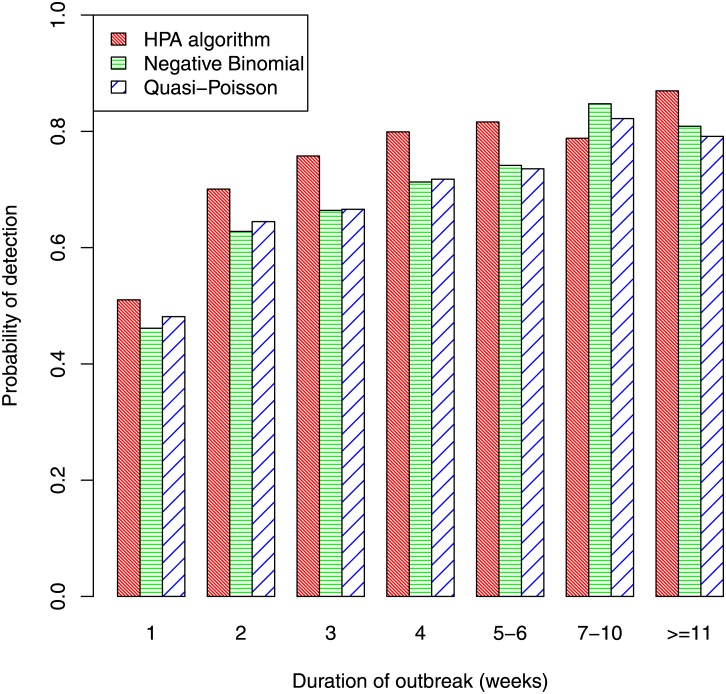
Probability of detection by duration of outbreak.

Turning to sensitivity, long outbreaks are easier to detect than short outbreaks, so sensitivity will vary with outbreak duration. Fig 6 shows the proportion of outbreaks that were detected by each algorithm, classified by outbreak duration. This proportion is only about 50% for outbreaks that lasted only one week, increases substantially for those lasting two weeks (the proportion was 70% for the HPA algorithm), and generally continued to increase as the duration of the outbreak increased. The overall probability of detection was 57% for the HPA algorithm, 51% for the negative binomial and 53% for the quasi-Poisson. There were 233 outbreaks of 7 or more weeks duration and, for these, the probability of detection was 83%, 83% and 81% for the HPA, negative-binomial and quasi-Poisson algorithms, respectively.

**Fig 7:**
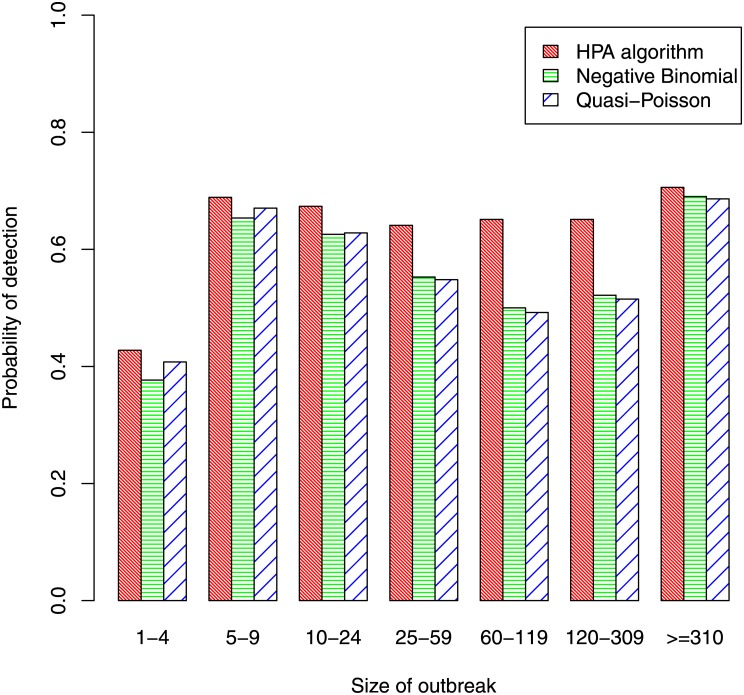
Probability of detection by size of outbreak.

Fig 7 shows the proportion of outbreaks that were detected by each algorithm, this time classified by the size of the outbreak. It shows that, for all three algorithms, sensitivity improves substantially as outbreak size increases from 1-4 cases to 5-9 cases. Thereafter, the level of sensitivity is fairly flat for the HPA algorithm but, somewhat surprisingly, for the other two algorithms it initially declines as outbreak size increases, only increasing again for the category containing the largest outbreaks. The reason is that, in general, larger outbreaks tend to occur in diseases that have the larger baseline rates and higher levels of background noise, making an outbreak more difficult to detect. Values on the ScPOD measure were calculated so as to obtain an overall measure of sensitivity that gave greater importance to larger outbreaks. Its value was 70.7% for the HPA algorithm, somewhat better than the values of 67.3% and 67.4% for the quasi-Poisson and negative-binomial algorithms.

**Fig 8:**
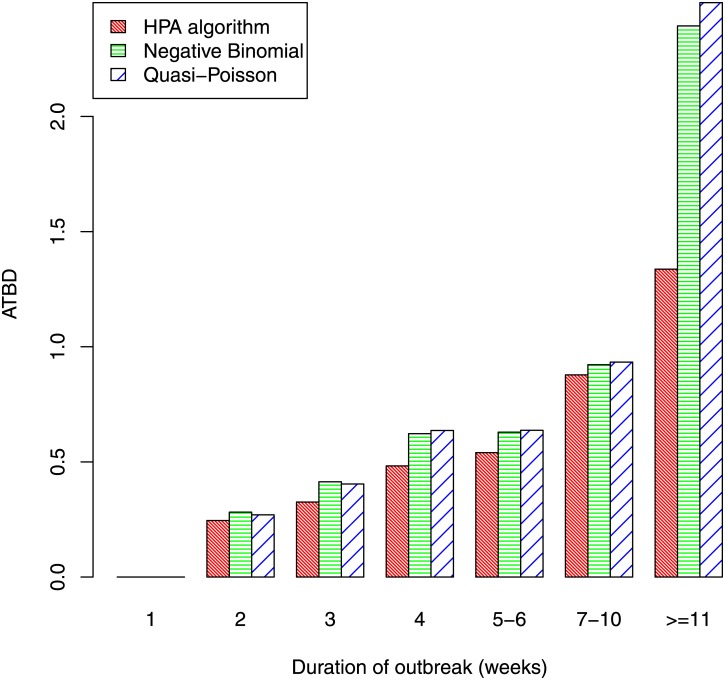
Average time before detection by duration of outbreak.

**Fig 9:**
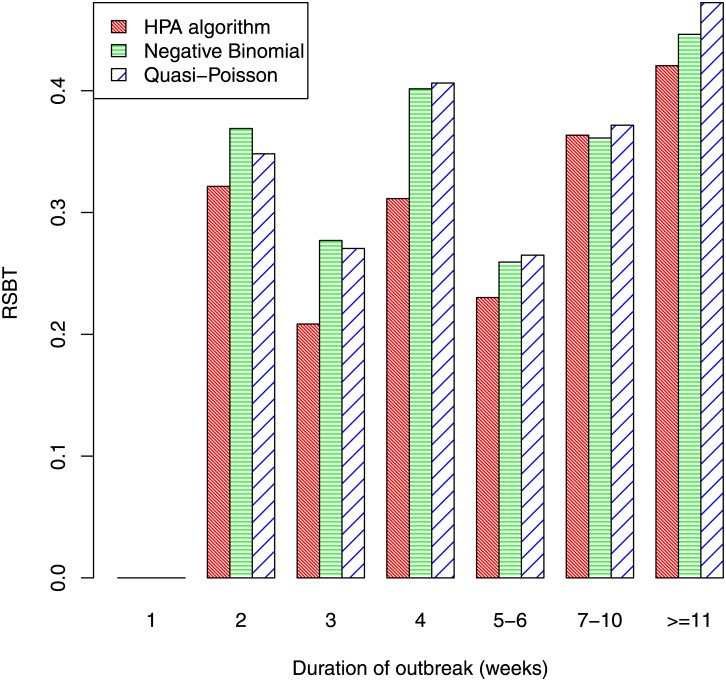
Relative size before detection by duration of outbreak.

Rather than simply detecting outbreaks, it is important to detect them in a timely fashion. Fig 8 indicates the weeks that elapsed before an outbreak was detected. We only consider outbreaks that were at some stage detected, as sensitivity has already been examined (Figs 6 and 7). Indeed, we only consider those outbreaks that were detected by all three algorithms, so that comparison of the algorithms is fair. The figure shows that outbreaks of less than 10 weeks duration were detected quickly (assuming they were detected); the delay was less than one week on average, and was similar for all three algorithms. For the longer outbreaks of 11 weeks or more, the HPA algorithm had a slightly longer average time before detection (ATBD) of 1.3 weeks, while the ATBD for the other two algorithms were more than a week poorer, at over 2.4 weeks. The relative size before detection (RSBD) is plotted against outbreak duration in Fig 9. As the HPA algorithm has a higher sensitivity than the quasi-Poisson and negative binomial algorithms, it would also be expected to have a better RSBD. The figure clearly shows this is the case, though differences between the three algorithms are small.

The beta scoring rule was used to make comparisons between algorithms that reflect both specificity and sensitivity. Table 2 gives the scores for each algorithm. The HPA algorithm has lower scores than the negative and quasi-Poisson algorithms in weeks without outbreak, but better scores when there was an outbreak. This is consistent with results presented earlier that showed the HPA algorithm has poorer specificity than the other two algorithms (Figs 4 and 5) but better sensitivity (Figs 6 and 7). Overall, the last column of the table shows that the newer algorithms had better beta (40, 0.5) scores than the HPA algorithm, indicating that they assess the probability of an outbreak better than the HPA algorithm. The scores of the two newer algorithms were quite similar for both outbreak and non-outbreak weeks, with the quasi-Poisson having slightly the better overall score. This latter result was a surprise—the quasi-Poisson algorithm is faster than the negative binomial algorithm but uses approximations that the negative binomial does not require.

**Table 2:** Scores of each algorithm for weeks with an outbreak, without an outbreak, and overall.

Algorithm	Weeks with an outbreak	Weeks without an outbreak	Overall
HPA	49 646	374 200	423 846
Negative binomial	34 730	408 621	443 351
Quasi-Poisson	32 697	411 241	443 938

**Fig 10:**
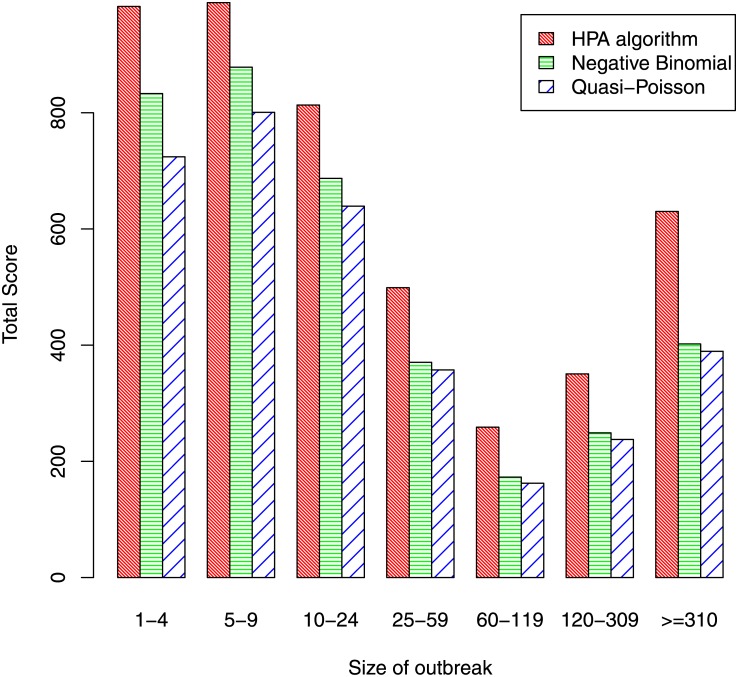
Scores by size of outbreak.

Fig 10 breaks down the scores for weeks with an outbreak according to the size of the outbreak. It can be seen that the relative scores of the different algorithms change little as the outbreak size changes. As was intended, outbreaks of all sizes make a substantial contribution to the overall scores of the algorithms.

The comparison of algorithms provided by a scoring rule is important. For this reason, the sensitivity of conclusions to the precise form of the scoring rule should be examined. To this end, we also evaluated the algorithms using two other forms of the scoring rule:

*Scoring rule 2*. The scoring rule parameters, *a* and *b*, were set equal to 31 and 1.5, so that *F*_1_ and *F*_2_ in Eq (7) are the cumulative distribution functions of beta(31, 2.5) and beta(32, 1.5) distributions, respectively. With this rule, the occurrence of an outbreak gives a higher score when *p* exceeds 0.95 and non-occurrence gives a higher score if *p* is less than 0.95. (The comparable balance-point for the beta (40, 0.5) scoring rule is 0.985, as noted earlier.)
*Scoring rule 3*. An unweighted form of the scoring rule in which the *g*_*i*_-weights in Eq (7) are fixed at unity. (As with the original scoring rule, *F*_1_ and *F*_2_ are the cumulative distribution functions of beta(40, 1.5) and beta(41, 0.5) distributions.)

Results for these scoring rules are given in Table 3. It can be seen that with both rules the quasi-Poisson algorithm is again marginally better than the negative binomial algorithm and both are noticeably better than the HPA algorithm. Hence the comparison of these algorithms shows some robustness to the choice of scoring rule.

**Table 3:** Overall scores given to algorithms by scoring rules 2 and 3.

Algorithm	Weighted beta(31, 1.5)	Unweighted beta(40, 0.5)
HPA	406 243	577 600
Negative binomial	439 998	580 560
Quasi-Poisson	441 136	581 274

## 6 Concluding comments

The study reported here followed the straightforward path of

1. constructing realistic series of test data in which it is known where outbreaks had been injected;
2. selecting/forming suitable evaluation metrics;
3. applying algorithms to the series of test data and comparing their performances using the evaluation metrics.

The test data were formed from time series of the actual weekly counts of the number of cases of individual diseases. The series for each disease was separated into a series of baseline counts (series *A*) and a series of outbreak counts (series *B*). The outbreak counts were translated by twelve months (giving series *C*) and recombined with *A* to form a test data series. This was done for 2304 different diseases, giving test data that reflected the diversity of patterns found in real data. The critical step in constructing the test data is the separation of the original series into baseline counts and outbreak counts. A number of methods were tried (not reported here) and their resulting time series of baseline and outbreak counts for individual diseases were examined. With the method that was adopted, visual inspection of plots showed that weeks with/without outbreak appeared to be classified sensibly.

As well as standard metrics, such as sensitivity and specificity, we also used a scoring rule to evaluate the performances of outbreak surveillance algorithms. Scores were derived from algorithms’ estimates of $p_{ij}^{*}$, the realistic probability that an outbreak has occurred in week *i* for disease *j*. Better estimates of $p_{ij}^{*}$ can simultaneously improve both sensitivity and specificity; otherwise sensitivity is normally only improved at the expense of reduced specificity, or vice-versa. With the scoring rule we used, the precise value of an estimate only matters when that value is above 0.9, which mirrors the range of values in which consideration should be given to flagging an outbreak and/or triggering an alarm. The scoring rule also has the useful property that larger outbreaks receive greater weight in the overall scores.

The results of our analysis show clearly that the HPA algorithm has slightly better sensitivity than the algorithms designed to replace it; the value of ScPOD was 70.7% for the HPA algorithm compared with 67.3% and 67.4% for the quasi-Poisson and negative binomial algorithms. Consistent with this finding, the HPA algorithm also had a shorter average delay time before outbreak detection and a smaller relative size before detection. However, the motivation for replacing the HPA algorithm was to improve specificity—a report on the HPA algorithm identified a high false positive rate as a limitation to its practical usefulness [2]. In that respect, the two new algorithms are highly successful; the overall false positive rates for the quasi-Poisson and negative binomial algorithms were 0.76% and 0.87%, respectively, which are approximately half the HPA rate of 1.63%. Pragmatically, these large reductions more than compensate for the slightly poorer sensitivity of the new algorithms. The scoring rule showed that the new algorithms also assess the probability of an outbreak better than the HPA algorithm, which on its own is reason to prefer them. In all respects the performances of the quasi-Poisson and negative binomial algorithms were very similar.

The purpose of this study was to compare three specified algorithms and this led to some limitations. In particular, the approach for generating test data is similar in many ways to the algorithms being considered. While this does not favour any one of the algorithms over the other two, it would become important if fundamentally different surveillance algorithms had to be evaluated for comparative purposes. In that case the mechanism for generating the test data would favour the HPA, negative binomial and quasi-Poisson algorithms. This could be avoided by altering Steps 2-4 in the test data generation process: the quasi-Poisson model in Step 2 might be replaced with a non-parametric smoother and non-parametric estimates of quantiles could be used in Steps 3 and 4. Another consequence from focusing on these three algorithms is that only *aggregated* counts across England and Wales were analysed. Local or small outbreaks that are confined, say, to a village or town will often be overlooked, while some spatiotemporal surveillance methods, such as SatScan [15], are designed to identify such local outbreaks. Broad reviews of outbreak surveillance methods are given in [3], [7] and [16].

In this paper, an outbreak has been defined as the addition of any cases to the baseline count. In practice, not all excesses would be classified as ‘outbreaks’ by subject-matter experts. Public Health England, for whom the algorithms were developed, use an algorithm to sort through hundreds of organisms each week and provide a shortlist of potential problems to be discussed by a team of epidemiologists. Their response to an abnormally high count will depend on many factors, most notably the disease, the assessed probability of an outbreak, and the estimated excesses in the current and preceding weeks. A typical response to an outbreak alert for a food or waterborne pathogen is to trace the source of contamination and remove it. For pathogens that are transmitted person to person, an outbreak may signal a problem with vaccine efficacy, or a change in pathogenicity.

The measures that are taken depend on context and some genuine outbreaks are not investigated at all. Hence, although the results from outbreak investigations could, in principle, be used to evaluate the accuracy of surveillance algorithms, selection biases would be hard to mitigate. Also, the results from an investigation are not always clear-cut, as not all investigations of common source outbreak find the common source and, if an outbreak is declared to be over (no further incident cases), the investigation will often be terminated without identifying causal exposure. In addition, some outbreaks are missed, as is clear from work reported in [17], who applied our negative binomial algorithm retrospectively to a laboratory database to look for two pathogens. They detected three outbreaks in a one-year period that were not previously recognised.

The study reported here is one of the most realistic evaluations of disease surveillance systems to have been conducted. It was possible because of the large reservoir of past data that was available. The original algorithm (the HPA algorithm) was in operation for over twenty years, monitoring infectious disease incidence for a population of 57 million, so decisions on its replacement could not be taken lightly. During the preparation of this paper, Public Health England have replaced the HPA algorithm with the negative binomial algorithm, in line with the findings reported here. As well as monitoring combined disease surveillance data from England and Wales, the new algorithm has also been applied to individual hospital trusts to examine time series of cases of important antimicrobial resistant pathogens. Modified forms of the new algorithms are also in use in the Robert Koch Institute, Berlin [18].

## Appendix A

Let {*y*_*i*_, *i* = 1, 2, …} denote the time-series of the number of cases of the disease under consideration, where *y*_*i*_ denotes a count in week *t*_*i*_. Let *t*_0_ denote the current week. The HPA algorithm uses quasi-Poisson regression to identify aberrations in the weekly counts. It assumes that *y*_*i*_ follows a linear time trend and is distributed with mean *μ*_*i*_ and variance *ϕμ*_*i*_:

$$\begin{array}{c} \text{log}\left(\mu_{i}\right)=\theta +\beta t_{i} \end{array}$$ (10)

where *ϕ* denotes a parameter that allows for over-dispersion. The following gives details.

1. The algorithm implicitly adjusts for seasonal effects by basing calculation of the expected count in the current week or just those counts that were observed in comparable weeks in the past. This approach also moderates the effects of events that occur at regular times each year, such as Christmas and summer holidays, which can affect when cases of diseases are reported. If *t*_0_ is week *τ* of the current year, then only data from weeks *τ* − 3 to *τ* + 3 of previous years are used in the analysis. Let *t*_(1)_,…, *t*_(n*)_ denote the weeks that are used and *y*_(1)_,…, *y*_(n*)_ the corresponding counts.
2. An iterative reweighting procedure is used to estimate model parameters and correct for past outbreaks in the baseline data. If there is no evidence of a linear time trend at the 5% significance level, then *β* is set equal to 0 in Eq (10). At convergence of the procedure, let ${\hat{\mu }}_{i}$ denote the estimate of *y*_(*i*)_ and let *w*_*i*_ be the weight in week *t*_(*i*)_ (*i* = 1,…, n*). The algorithm’s estimate of *ϕ* is
  $$\begin{array}{c} \hat{\phi }=\max\left\{\frac{1}{n^{*}-r}\sum_{i=1}^{n^{*}}w_{i}\frac{{\left(y_{\left(i\right)}-{\hat{\mu }}_{i}\right)}^{2}}{{\hat{\mu }}_{i}},\;1\right\} \end{array}$$ (11)
  where *r* = 1 or *r* = 2 depending on whether a time trend has been fitted. The weights satisfy
  $$\begin{array}{c} w_{i}=\left\{\begin{array}{cc} \gamma s_{i}^{-2} & \;\text{if}\;s_{i}>1\\ \gamma & \;\text{otherwise} \end{array}\right. \end{array}$$ (12)
  where *γ* is a constant such that $\sum_{i=1}^{n^{*}}w_{i}=n^{*}$ and the *s*_*i*_ are scaled Anscombe residuals,
  $$\begin{array}{c} s_{i}=\frac{3}{2{\hat{\phi }}^{1/2}}\frac{y_{\left(i\right)}^{2/3}-{\hat{\mu }}_{i}^{2/3}}{{\hat{\mu }}_{i}^{1/6}{\left(1-h_{ii}\right)}^{1/2}}, \end{array}$$ (13)
  where *h*_*ii*_ are the diagonal elements of the hat matrix.
3. Let ${\hat{\mu }}_{0}=\hat{\theta }+\hat{\beta }t_{0}$ denote the expected value of *y*_0_, the count for the current week. The algorithm calculates the threshold value, *U*, from
  $$\begin{array}{c} U={\hat{\mu }}_{0}{\left\{1+\frac{2}{3}z_{\alpha }{\hat{\mu }}_{0}^{-1}{\left(\hat{\phi }{\hat{\mu }}_{0}+\text{var}\left({\hat{\mu }}_{0}\right)\right)}^{1/2}\right\}}^{3/2} \end{array}$$ (14)
  where *z*_*α*_ is the 100(1 − *α*)-percentile of the standard normal distribution. Applying a 2/3 power transformation to a Poisson variate induces an approximately symmetric distribution, which underlies the 3/2 power in Eq (14).
4. The exceedance score is then given by $X=(y_{0}\;-\;{\hat{\mu }}_{0})/(U-{\hat{\mu }}_{0})$, as in Eq (1).

The quasi-Poisson and negative-binomial algorithms model seasonality through a 10-level factor that has a 7-week reference period (corresponding to weeks *τ* ± 3, as in the HPA algorithm) and nine 5-week periods each year:

$$\begin{array}{c} \text{log}\left(\mu_{i}\right)=\theta +\beta t_{i}+\delta_{\tau (t_{i})} \end{array}$$ (15)

where *δ*_*τ*(*t*_*i*_)_ is the seasonal factor. These algorithms always include a term for linear trend (never setting *β* to 0), and only set *w*_*i*_ equal to $\gamma s_{i}^{2}$ when *s*_*i*_ > 2.58, rather than *s*_*i*_ > 1 (c.f. Eq (12)). The quasi-Poisson model calculates the threshold value as in Eq (14), while the negative binomial algorithm calculates it under the assumption that, when there is an outbreak, the number of cases above baseline in a week follows a negative binomial distribution.

## Supporting Information

S1 R.Codes S1_R.Codes.R: An R code used in the implementation of test data and comparison of statistical algorithms. (R)
